# Peroxisome Proliferator-Activated Receptors (PPARs) and Oxidative Stress in Physiological Conditions and in Cancer

**DOI:** 10.3390/antiox10111734

**Published:** 2021-10-29

**Authors:** Giuliana Muzio, Giuseppina Barrera, Stefania Pizzimenti

**Affiliations:** Department of Clinical and Biological Sciences, University of Turin, Corso Raffaello 30, 10125 Turin, Italy; giuseppina.barrera@unito.it (G.B.); stefania.pizzimenti@unito.it (S.P.)

**Keywords:** peroxisome proliferator-activated receptors (PPARs), oxidative stress, cancer, inflammation

## Abstract

Peroxisome proliferator-activated receptors (PPARs) belong to the nuclear hormone receptor superfamily. Originally described as “orphan nuclear receptors”, they can bind both natural and synthetic ligands acting as agonists or antagonists. In humans three subtypes, PPARα, β/δ, γ, are encoded by different genes, show tissue-specific expression patterns, and contribute to the regulation of lipid and carbohydrate metabolisms, of different cell functions, including proliferation, death, differentiation, and of processes, as inflammation, angiogenesis, immune response. The PPAR ability in increasing the expression of various antioxidant genes and decreasing the synthesis of pro-inflammatory mediators, makes them be considered among the most important regulators of the cellular response to oxidative stress conditions. Based on the multiplicity of physiological effects, PPAR involvement in cancer development and progression has attracted great scientific interest with the aim to describe changes occurring in their expression in cancer cells, and to investigate the correlation with some characteristics of cancer phenotype, including increased proliferation, decreased susceptibility to apoptosis, malignancy degree and onset of resistance to anticancer drugs. This review focuses on mechanisms underlying the antioxidant and anti-inflammatory properties of PPARs in physiological conditions, and on the reported beneficial effects of PPAR activation in cancer.

## 1. Introduction

The maintenance of intracellular redox homeostasis is crucial to ensure functionality and survival of normal cells. Antioxidant defenses include several enzymes and molecules responsible for the cellular response to oxidative stress. The expression of several of these molecules is directly or indirectly regulated by transcription factors, including Peroxisome Proliferator-Activated Receptors (PPARs). In this view, PPAR antioxidant properties have been deeply investigated in both physiological and pathological conditions. This review focuses on the mechanisms regulating PPAR expression and activity in normal cells, paying particular attention on their involvement in controlling oxidative stress and inflammation. Moreover, knowledge on the effects of PPAR-mediated modulation of oxidative stress in cancer development and progression is also summarized.

## 2. PPAR Physiology

PPARs belong to nuclear hormone receptor superfamily and share characteristic functional domains with other superfamily members. Originally described as “orphan nuclear receptors”, PPARs are now known to bind a multitude of both natural and synthetic ligands acting as agonists, antagonists, or inverse agonists [1,2,3,4]. Since several of these ligands share the ability to induce, with different entity, peroxisome proliferation in rodent hepatocytes, their receptors have been indicated as PPARs.

In humans three different subtypes, PPARα, β/δ, γ, are encoded by separate genes and show tissue-specific expression patterns [5]. From *PPARγ* gene three different mRNA are transcribed (γ1, γ2, γ3) leading to the production of two proteins. All subtypes are involved in the regulation of lipid and carbohydrate metabolisms (Table 1); moreover, based on their expression in specific cell types or tissues, PPARs can play a pivotal role in modulating different cell functions (proliferation, death, differentiation) [6], inflammatory process [7], angiogenesis [8], immune response [9].

The ligand-dependent biological effects of PPARs require their heterodimerization with the nuclear receptor Retinoid X Receptor (RXR) and a conformational change that determines co-repressor release and co-activator recruitment. In this activated form, the heterodimer PPAR/RXR binds to protein complexes, including histone acetyltransferase, and to RNA polymerase II, this allowing chromatin remodeling, recruitment of transcription machinery and transcription of target genes (trans-activation action) [14].

In these genes, the DNA-binding sequence of PPAR/RXR complex is called Peroxisome Proliferator Response Elements (PPRE) and consists of a direct repeat (DR-1) motif composed of two half-sites that occur as a direct repetition of a hexanucleotide DNA sequence (AGGTCA) with a single nucleotide spacing between the two repeats. PPRE can be located within promoter, or introns/exons or in 3′ downstream region of target genes [15].

The expression of PPARs has been recently evidenced to be epigenetically modulated by a large number of miRNAs that target the different isoforms. This type of posttranscriptional regulation has been characterized in different diseases in which the expression of specific miRNA contributes to the pathogenesis, mainly through the downregulation of PPARs [16,17,18]. In a recent review, Sundrani et al. [19] suggested that fatty acid-mediated alterations in miRNAs/PPARs axis could be responsible for placental disorders leading to babies with low birth weight. In a rat model of intestinal injury after hypoxia/re-oxygenation, the increased expression of miR-23a-5p has been reported to further worsen cell damage by decreasing PPARα and its antioxidant effect, this leading to an increased ROS production [20].

The ability of miRNAs in modulating PPAR expression has also been evidenced for the miRNAs delivered in the exosomes. In fact, miR-130a-3p, present in acinar cell-derived exosomes, has been demonstrated to contribute to pancreatic fibrosis onset by decreasing the PPARγ activity in stellate cells by directly binding to the PPAR-3′UTR [21]. miRNAs targeting both PPARγ and δ have been found to be hyper-expressed in macrophages isolated from visceral adipose tissues of obese mouse and contribute to the induction of insulin-resistance [22,23].

Other than in a direct way, miRNAs can also indirectly affect the PPAR expression/functions since several complexes regulating PPAR transcriptional activity (co-activators and co-repressors) are in turn targeted by specific miRNAs [24,25,26].

The importance of the axis miRNAs/PPARs in modulating different cell functions was further confirmed by the observation that PPRE sequences are present in the promoter of several miRNAs to indicate a mutual modulation [16].

As previously reported, the activity of PPARs is regulated at different levels: by several molecules or multiprotein complexes that can act as co-activators or co-repressors, and by different types of post-translational modifications.

PPAR co-activators are known to show different intrinsic biological activities. They include: (1) some enzymes, as histone acetylases (CBP/p300 and steroid receptor coactivator, SRC-1) and ATPases (Brg1 or Brm members of SWI/SNF complex), responsible for chromatin remodelling; (2) proteins forming a bridge between the nuclear receptor and the transcription initiation machinery (PPAR binding protein/thyroid receptor associated protein 220, PBP/TRAP220) [27,28,29,30].

Peroxisome proliferator-activated receptor gamma co-activator 1 alpha (PGC-1), originally identified as a specific co-activator of PPARγ and now known to modulate the activity of several transcription factors, favours PPAR/RXR action recruiting other co-activators provided with histone acetyltransferase activity, and induces RNA polymerase II action directly interacting with the TRAP/DRIP [31,32,33,34].

PPARs can heterodimer with RXR also in the absence of ligands and in this form the dimer remains tied to the co-repressor complexes, such as NCoR (Nuclear receptor corepressor), SMRT (Silencing mediator of retinoid and thyroid hormone receptor) or RIP140 (Receptor Interacting Protein 140). These complexes directly or indirectly recruit histone deacetylases (HDACs) that repress gene transcription via modulation of chromatin structure [35,36,37].

PPAR activity is also modulated by different types of post-translational modifications, named PTM, that include phosphorylation, SUMOylation, ubiquitination and acetylation. The studies examining this aspect of PPAR physiology have been mostly conducted on PPARα and γ. This type of regulation is very articulated, since the PTM effects on PPAR activity depend on various factors, as the PPAR isoform, the site of modifications, the effector of PTM and the substrate availability.

The phosphorylation of PPARα and γ occurs at specific serine residues and can be due to MAPK, CDK or GSKβ. PPARα phosphorylation in serine 12 and 21 by MAPK or CDK7 is known to increase its activity possibly via improved recruitment of co-activators and release of co-repressors; differently, the degradation of PPARα is favoured by GSKβ-mediated phosphorylation at serine 73 [38]. With regard to PPARγ, the phosphorylation of serine 112 determines a different effect (activation or repression) depending on the kinase involved: the activity is increased in case of MAPK-mediated phosphorylation, whereas is reduced in case of CDK7/9 involvement [39,40,41,42].

SUMOylation negatively regulates both PPARα and γ [43,44,45]. SUMOylation of lysine 185 or 358 in PPARα decreases its activity in hepatic cell lines and in liver, mainly through an increased binding to NCoR co-repressor [46,47]. In PPARγ, several putative SUMOylation sites have been identified [48], but mainly the effects on serine 107 have been extensively investigated [39,49]. The importance of PTM in regulating PPARγ activity has been further confirmed by the observation that the phosphorylation at serine 112 seems to favour the SUMOylation at lysine 107 [49,50], suggesting a possible interconnection among the different PTM pathways.

Polyubiquitination of all PPAR isoforms causes a decrease of transcriptional activity due to their proteasomal degradation [51,52,53,54].

### PPARs, Inflammation and Oxidative Stress

All PPAR isoforms hare well known to possess anti-inflammatory activity that takes place through different mechanisms that are summarized in the Table 2.

The ability of PPARs in decreasing the synthesis of pro-inflammatory mediators in several pathological conditions and in inflammatory experimental models has been partially attributed to the ligand-dependent [trans-repression of transcription factors, mainly NF-kB, AP-1 and STAT. NF-κB transcriptional activity is inhibited by a direct interaction of all PPAR isoforms to its p65 component [55,56,57,58]. Other than by binding to the ligands, the PPAR-p65 interaction seems to be also modulated by MAPK signalling since ciglitazone induces MAP kinase-mediated phosphorylation of PPARγ, this leading to a major inhibition of NF-kB [55].

PPARα ligands, fibrates and WY-14643, have been evidenced to further decrease NF-kB pathway via modulation of the expression, stability or activity of the inhibitor IkB-α, [56,59,60].

**Table 2:** Principal mechanisms responsible for the anti-inflammatory activity of PPARs.

Mechanism	Characteristics/Effects	Isoform	References
Ligand-dependent trans-repression activity	(1) independent from the binding to specific DNA sequences (2) based on the interaction between ligand-bound ppar and components of other transcription factors (NFKB, AP-1) or regulatory complexes (iNOS) (3) based on the inhibition of kinases involved in the activation of other transcription factors	α, β/δ, γ	[61,62,63,64,65,66,67,68]
Modulation of inflammasoma activity	(1) decreased expression of NLRP3, CASPASE 1, IL-1β, IL-18 (2) decreased expression of TLR	α, β/δ, γ	[69,70] [71,72]
Modulation of pro-inflammatory genes	(1) increased expression of anti-inflammatory miRNA (miR-142-3p, miR-124)	γ	[73,74]
Direct up-regulation of genes with anti-inflammatory properties.	(1) increased expression via binding to ppre in target genes (HO-1, C3, PGlyRPs, IL-10, eNOS, UCP2)	α	[75,76,77,78]
crosstalk with NRF2	(1) “ARE” Sequences Present in PPARγ promoter (2) putative PPRE sequences IN Nrf2 gene	γ	[79,80] [81]

A direct protein-protein interaction is also responsible for the trans-repression of AP-1 activity; in fact, GST pull-down experiments carried out by Delerive et al. [81] evidenced a binding of PPARα to the amino-termi nal domain of c-Jun.

The transcription of STAT5 target genes has been reported to be inhibited in a dose-dependent way by PPARα and γ activation [82]. This observation evidenced a reciprocal negative regulation between PPAR and STAT pathways, since it was previously demonstrated that STAT5b inhibits PPAR transcriptional activity by binding to ligand–independent AF-1 domain in PPARs [83,84]. The lack of STAT5b inhibition evidenced in case of PPARα lacking AF-1 confirmed the importance of this domain in STAT5b-PPAR crosstalk. The ability of PPARs in reducing STAT pathway has also been confirmed by the finding that in human macrophages PPARα and γ agonists decreased STAT-mediated transactivation without directly decreasing the DNA binding of these transcription factors [85].

More recently, the treatment with HuoXueTongFu Formula, a liquid herbal formula used in traditional chinese medicine and possessing anti-inflammatory properties, has been shown to prevent intraperitoneal adhesion occurring after abdominal surgery by modulating cytokine production via PPARγ-mediated downregulation of SOCS3/JAK2/STAT1 pathway [86].

Over the past 20 years, the ability of PPARs in modulating inflammasome activity was included among the mechanisms responsible for their anti-inflammatory properties. In particular, the majority of the studies investigated PPARs as regulators of NLRP3, the most studied inflammasome complex [74].

PPARγ activation via both natural and synthetic ligands, ω-3 polyunsaturated fatty acids, (PUFA) and rosiglitazone, have been demonstrated to inhibit NLRP3 increased transcription occurring in mice deficient for CGI-58, a co-activator of adipose triglyceride lipase, administered with a lipid rich diet, and in LPS-treated murine Raw 264.7 cells [68,87]. The beneficial effect of Morin (3,5,7,2′,4′-pentahydroxyflavone), a natural flavonoid, on obesity induced by high-fat diet in rats has been ascribed to its binding to PPARα and the consequent decrease of mRNA of the NLRP3 inflammasome complex that is overexpressed in this pathological condition. Moreover, the PPARα activation positively affects fatty acid catabolism improving lipid profile in liver. A similar effect, albeit to a lesser extent, has been observed in the same experimental model after treatment with a mixture of EPA and DHA (eicosapentaenoic and docosaexaenoic acid, respectively) [88]

The efficacy of rosiglitazone in reducing inflammation via modulation of NLRP3 has been more recently confirmed in a model of radiation-induced acute intestinal injury and in intestinal macrophages isolated from the same animals [69].

In a similar way, the anti-inflammatory activity showed by abscisic acid, a natural phytohormone, in a murine model of allergic airway inflammation has been attributed to its ability in increasing PPARγ expression and, in consequence, in inhibiting NLRP3 [89]. In a mouse model of NAFLD (Nonalcoholic fatty liver disease), GW501516, a specific PPARβ/δ ligand, inhibited the activation of NLRP3, NLRP6, and NLRP10 and decreased the production of pro-inflammatory molecules induced by high fat diet and LPS [90].

At the present, two mechanisms have been suggested as responsible for the PPARγ-mediated modulation of inflammasome activity: a downregulation of the expression of inflammasome components and a direct interaction between DBD domain of PPAR and NLRP3, this interfering with NLRP3 assembly [69,91].

The crosstalk between PPARs and Nrf2 (NF-E2-related factor 2), has been evidenced to play an important role not only in modulating inflammatory process, but also oxidative stress. Nrf2 is considered as one of the most important regulator of cellular response to oxidative stress [92,93,94] and of xenobiotic metabolism, via binding to Antioxidant Response Element (ARE) in the promoter of target genes. The anti-inflammatory action of Nrf2 is mainly due to its ability in modulating several pathways and determining: (1) inhibition of NF-kB transcriptional activity via competition with co-activator CBP [95,96,97]; (2) inhibition of the expression of cytokines TNFα, IL-6, IL-1β, and of COX and iNoS [98,99,100].

The involvement of both Nrf2 and PPARs in modulating inflammatory process suggested the possibility of a mutual control of the expression of these transcription factors. This hypothesis has been directly confirmed by the observations that two ARE are present in PPARγ promoter [78,79] and a putative PPRE is present in Nrf2 promoter region [101]. Based on these observations several studies investigated the possibility to improve the regulation of inflammation process by acting simultaneously on PPAR and Nrf2 pathways. With this aim, the effects of specific Nrf2 or PPAR activators, or dual agonists have been investigated in both in vivo and in vitro models of different diseases characterized by increased inflammation and oxidative stress. The results evidenced, in most cases, greater beneficial outcomes and confirmed the significant crosstalk between PPARs and Nrf2 [102,103,104]. This last aspect has been further underlined by the observation that the promoter of some genes, as glutathione transferase, contains both ARE and PPRE sequences [101].

PPAR anti-inflammatory properties also contribute, in an indirect way, to their ability in acting as regulator of cellular response to oxidative stress. As a whole, the antioxidant activity of PPARs is mainly due to their ability in directly activating the transcription of genes showing antioxidant activity, in modulating at post-transcriptional level the activity of proteins with antioxidant functions, or negatively affecting the expression/activity of proteins generating or metabolizing free radicals.

Table 3 summarises genes containing PPRE in their promoter region, as evidenced by bioinformatic approach or reporter gene assay.

The presence of the PPAR DNA-binding sequence in genes under transcriptional control of PPARs has been previously suggested by studies using specific ligands for the different PPAR isoforms, knockout animals or silencing technique.

The researches on this topic deeply investigated the correlation between PPARγ and catalase expression, evidencing that this isoform prevents, via catalase induction, the oxidative stress associated with different conditions, as apoptosis induction [117], pesticide inhalation [118], LPS-induced brain impairment [119], hypertension [120], calcium oxalate-induced nephrolithiasis [114]. This antioxidant effect has been observed using canonical synthetic PPARγ agonists (pioglitazone, rosiglitazone) [119,121], natural ligands, as catalpol [122] or a combination of synthetic and natural ones (carvacrol plus pioglitazone [118].

In a similar way, PPARα agonist fenofibrate has been recently reported to reduce oxidative stress through the increased expression of catalase in cultured rat cardiomiocytes exposed to high glucose uptake or hypoxia/reperfusion condition [123]. The fenofibrate-mediated increase of catalase expression, and the consequent improvement of redox state, also occurred in testicular tissue of diabetic rats [124].

PPARβ/δ has been evidenced to play a crucial role in maintaining function and characteristics of vascular endothelium in in vivo and in vitro studies through its ability in modulating the expression of antioxidant enzymes, including catalase.

In a rat model of ischemia/reperfusion PPARβ/δ activation with the specific ligand GW0742 upregulated catalase, UCP-3 and superoxide dismutase 2 (SOD2) [125]. The consequent reduction of oxidative stress has been demonstrated by the decreased production of 4-hydroxy-2-nonenal (HNE), the major lipid peroxidation product, and of formation of its adducts with proteins. Interestingly, this research also evidenced that PPARβ/δ increased at transcription level the expression of aldehyde dehydrogenase 2 (ALDH2), the mitochondrial isoenzyme of ALDH known to metabolize acetaldehyde and HNE derived from lipid peroxidation [126,127]. This observation indicates that PPAR modulation of oxidative stress is also due to their ability in favouring the metabolism of highly reactive aldehydic products of lipid peroxidation.

As in the case of catalase, also SOD expression is directly increased by ligands of all PPAR isoforms. This contributes to the beneficial effects of these molecules reported in different pathological conditions characterized by oxidative stress, including oxidative damage of pancreatic cells and atrial remodelling in diabetes [128,129], intervertebral disc degeneration responsible for of back and neck pain [130], benign prostatic hyperplasia [131].

An important crosstalk involved in intracellular defence against oxidative and inflammatory conditions concerns PPARs and heme oxygenase (HO), mainly HO-1. All PPAR isoforms have been reported to induce the expression of HO-1 [132]. PPAR-mediated expression of HO-1 contributes in preserving/improving vascular integrity in different conditions of vascular disorders, as angiotensin II-induced hypertension in rats [133,134] and pulmonary hypertension due to proliferation of smooth muscle cells [135]. In iron-challenged hepatocytes, the adiponectin-mediated activation of axis PPAR/HO-1 decreases apoptotic cell death and iron accumulation, reducing the clinical hepatic consequence of iron overload [136]. A similar protective effect due to PPARγ and HO-1 overexpression/activation has been described in an animal models of fibrotic steatohepatitis [137] and in renal damage consequent to ischemia/reperfusion [138]. The ability of HO in influencing in turn PPAR activity has been documented by the observation that in macrophages and muscle cells carbon monoxide produced by HO-1 activity is able to increase PPARγ and β/δ, this leading to beneficial effect through the modulation of different signalling transduction pathways, including inhibition of early growth response-1 (Egr-1) and activation of ERK5 [139,140,141,142].

Uncoupling proteins (UCPs) contribute to the maintenance of intracellular redox homeostasis by decreasing the ROS production in mitochondria [143]. In this view, the regulation at transcriptional level of their expression by PPARs represents an important mechanism contributing to cellular defence toward oxidative stress. The first studies on this aspect of PPAR antioxidant activity mainly investigated the effect of PPARα and γ ligands on the expression and activity of the three UCP isoforms. Teruel’s group evidenced that in rat brown adipocytes rosiglitazone increased UCP-3 and UCP-1, and WY14643 increased UCP-3, also suggesting a possible synergic or competitive effect of contemporary administration of both ligands depending on the specific UCP isoform [114,115,144]. Transcriptional upregulation of UCP-2 induced by rosiglitazone protects against oxidative stress in rostral ventrolateral medulla, preserving the activity of premotor neurons responsible for the maintenance of neurogenic vasomotor tone, and reducing hypertension [145]. More recently, PPARβ/δ specific ligand GW0742 has been shown to relieve endothelial alterations induced by LPS via upregulation of UCP-2 and the consequent reduction of intra-mitochondrial ROS production [146].

## 3. PPARs and Oxidative Stress in Cancer

Based on the multiplicity of physiological effects above-described, PPAR involvement in cancer development and progression has attracted great scientific interest.

The main observation emerged from the studies is that a wide species specificity characterizes the effects of the activation of different PPAR isoforms on carcinogenesis process.

The species specificity of PPAR effects has been above all documented for PPARα whose activation, by natural or synthetic ligands, resulted associated with hepatocarcinoma occurrence in rodents, and not in other species. In sensitive species, PPARα activation seems to drive liver carcinogenesis through several mechanisms, including increase of cell proliferation via activation of c-Myc and cyclins, decrease of susceptibility to undergo apoptosis, increase of oxidative stress [147,148,149]. This last event seems to be triggered by the PPARα-mediated increase of enzymes showing oxidase activity and different subcellular localisation, as in peroxisome, endoplamatic reticulum, and mitochondria.

In particular, acyl-CoA oxidase (ACO) [150], NADPH oxidase [151,152] and cytochrome P4504A1 [153] have been indicated as responsible for the increased ROS production contributing to the initiation phase of carcinogenic process in murine liver. In the same animals, a PPARα-mediated energy burning in liver has been also indicated as a factor contributing to hepatocarcinogenesis [154].

In the various species, the different ability of PPARα ligands in inducing ROS formation has been related with a different basal expression level present in the hepatocytes. In particular, in human liver, a low level of PPARα mRNA and protein has been reported [155], even if this observation has not been confirmed in a study comparing the consequences of PPARα activation in cultured human and rodent hepatocytes, and suggesting a major effect of PPARα [156]. Other than to the low expression level, a different sequence in ligand-binding domain (LBD) of PPARα could decrease the response to the same ligand concentrations in primates and humans and, in consequence, their susceptibility to undergo carcinogenesis process.

With regard to the changes occurring in cancers and cultured cancer cells, both increase or decrease of PPAR expression have been reported (Table 4). Anyway, the alterations in the activity of these nuclear receptors always significant contribute to the acquisition of cancer phenotype.

The different trend in PPAR expression evidenced in various types of tumours could be related to the activity levels present in the corresponding normal cells or to the tissue-specific functions played by each isoform. Interestingly, a correlation between PPAR expression and malignancy degree have been observed in some tumours, this further underlying the pivotal role played by PPARs also in cancer progression and suggesting the possibility to different affect cancer cells with different malignancy degree using PPAR ligands [163].

In this view, conjugated linoleic acid (CLA) has been shown to be more effective against cancer cells with higher malignancy degree and originated from different tissues. Moreover, the kind of effect was dependent on the different modulation of PPAR isoforms. In fact, the contemporary increase of PPARα and decrease of PPARβ/δ was associated with the induction of apoptotic death, whereas PPARγ activation with the inhibition of cell proliferation [63]. These results have been confirmed in human hepatocarcinoma HepG2 cells where the specific PPARα agonist clofibrate caused apoptosis in a time- and concentration-dependent way, and PGJ2, a ligand of PPARγ, inhibited cell proliferation [173]. In rat hepatoma cell lines exposed to high clofibrate concentrations capable of also activating PPARγ, the inhibition of proliferation was mediated by the increase of protein phosphatase 2A and the consequent decrease of Erk1,2 and c-myc [174]. The increased expression of PP2A has been later attributed to the presence of putative PPRE sequences in genes encoding its subunits [175]. In human hepatocellular carcinoma HepG2 and in colorectal adenocarcinoma CaCo2 cells, the concurrent administration of PPARα ligand clofibrate and of JNK inhibitor AS60012145 reduced proliferation and induced apoptosis, likely via the overexpression of 28 genes containing PPRE [176]. PPAR ligands have been shown to reduce cancer cell growth also acting synergistically with HNE [177,178].

The anticancer effect of PPARs is also due to their ability in modulating oxidative stress, through the effect on the expression of antioxidant enzymes, the induction of lipid peroxidation and the activity of enzymes metabolizing the lipid peroxidation products.

These effects have been observed using both natural or synthetic ligands of the different isoforms.

With regard to the involvement of PPAR activation in early stages of carcinogenesis process, the beneficial reported effects can be mainly referred to the prevention of oxidative stress associated with tumoral cell transformation.

In animal experimental models of lung cancer, oral administration of rosiglitazone resulted in an increase of GPx expression and glutathione content, and in a decreased of malondialdeyde (MDA) production, this blocking the diethylnitrosamine-induced cancer formation [179]. In a similar way, Balupillai and coll. [180] demonstrated that the induction of photocarcinogenesis in mice skin was prevented by caffeic acid probably through the activation of PPARγ and the improvement of oxidative imbalance induced by UVB. Lycopene, a natural carotenoid known to prevent the development of several types of cancers, has been shown to activate PPARγ in esophageal cancer cell line EC109, and to inhibit, at appropriate doses, the esophageal carcinogenesis induced by methylbenzylnitrosamine in rat, via the reduction of oxidative stress-mediate MDA formation and of pro-inflammatory cytokine level [181,182].

Studies on cultured tumour cells, derived from different tissues, evidenced that the anticancer effect of PPARs can occur also through the modulation of different signalling pathways and the induction of oxidative stress causing a cytostatic or cytotoxic effect.

Arachidonic acid, a natural ligand of PPARs, caused suppression of growth of lung cancer cells A549, through the increase of PPARγ and lipid peroxidation. In this cells the known cytostatic effect of lipid peroxidation products [183] was augmented by the decreased expression of cytosolic ALDH3, the main responsible for the catabolism of aldehydes derived from PUFA oxidative breakdown. The reduction of ALDH3 expression has been demonstrated to be due to the PPAR-mediated inhibition of NF-kB, for which several consensus sequences are present in the ALDH gene [184].

In lung cancer cells A427, both n-3 and n-6 PUFA increased PPARα expression and lipid peroxidation, and inhibited cell proliferation [185]. In this research, a putative PPRE has been evidenced in genes encoding the fast isoforms of myosin heavy chain, which suggested the PPAR activation as possible therapeutic approach against cancer cachexia. To be noted that treating cancer cells with PUFA is motivated by the fact that carcinogenetic process is associated with a reduced PUFA amount in membrane phospholipids, this contributing to their decreased susceptibility to undergo lipid peroxidation [186,187].

The cytostatic effect of PPARs has been evidenced also by Srivastava and coll. [188] in lung adenocarcinoma cells NCI-H2347 and NCI-H1993, where an alternative pathway of PPAR-mediated cell growth inhibition has been documented. In fact, in this experimental model, the PPARγ activation by pioglitazione caused a metabolic switch consisting in a decrease of pyruvate oxidation and GSH level. In turn, the decreased intracellular antioxidant defence augmented ROS production determining the hypophosphorylation of retinoblastoma protein and the block of cell cycle.

Using a multi-tiered approach, Savic and coll. [189] confirmed in human colon cancer cells HT-29 that PPARγ activation decreases proliferation through the increase of lipid peroxidation consequent to the GSH reduction.

A mutual influence among PPAR isoforms has been observed in human squamous carcinoma cells SCC-15, where silencing PPARγ caused an increase of PPARα and β mRNA [190]. The authors suggested the possibility that PPARγ negatively regulates the expression of other PPARs. This finding could be important in designing new therapeutic approaches aiming to differently affect various PPARs in cancers.

An important event reducing the efficacy of anticancer therapies is the onset of cancer cell resistance to chemotherapeutic drugs, that can be due to several mechanisms, including the increase of antioxidant defences and drug transporter proteins, decrease of detoxifying enzymes or susceptibility to apoptosis.

The above-reported ability of PPARs in modulating ALDH expression, confirmed also in rat hepatoma cells and human breast cancer cells [191,192], can represent a strategy to reduce the resistance to anticancer drugs acting through the formation of free radicals, as doxorubicin, or oxazaphosphorines, whose metabolic intermediates are catabolized by ALDH [193]. It is important underlying that in several type of cancers the expression of ALDH1 and ALDH3 is constitutively increased and contributes to their decreased susceptibility to the effects of lipid peroxidation products. [194].

Superparamagnetic iron oxide nanoparticles (SPIONs) functionalized with CLA for theranostics use, other than to inhibit breast cancer cell growth via PPARγ activation, also decreased the expression of P-glycoprotein, a drug efflux transporter. This effect is probably the consequence of PPAR-mediated induction of necrosis, increase of TNFα and IL-1β, cytokines known to inhibit P-glycoprotein expression [192,195].

In gastrointestinal cancer cells, the cisplatin resistance, characterized by increased expression of aldo-keto reductase 1B10 (AKR1B10, an enzyme associated with resistance) against anticancer drugs and decreased PPARγ, is reverted by a combined treatment with a AKR1B10 inhibitor and rosiglitazone [196]. The onset of resistance to cisplatin reduces the therapy efficacy also against brain cancers. Human glioma cells U-87 overexpressing PPARγ showed a decreased production of P-glycoprotein, increased oxidative stress and sensitivity to cisplatin in comparison with parenteral ones [197].

The importance of PPARs in preventing the chemoresistance occurrence has been confirmed in non-small cell lung cancers (NSCLC) where hypoxia, via HIF-1, has been evidenced to contribute to the resistance to cisplatin or docetaxel. The mechanisms responsible seems to be the inhibition of PPARγ, the decrease of UCP-2, and the consequent ROS-induced expression of ABC transporter protein ABCG2, which causes drug efflux [198].

In the same type of cancer cells, CB13 (1-benzyl-5-(4-methylphenyl) pyrido [2,3-d]pyrimidine-2,4(1H,3H)-dione), a recently synthesized PPARγ ligand, reduces the radio-resistance via the production of ROS and induced apoptosis through the stimulation of caspase-3, and caspase-9 activities [199].

Since the adaptive increase of antioxidant defences in cancers represents an important factor in inducing chemoresistance, several studies evidenced that a constitutive activation of Nrf2, due to increased expression or mutations in Nrf2 or its inhibitor KEAP1, contributes to the resistance to therapy in various types of cancers [200].

In this view, the above-reported crosstalk between PPARs and Nrf2 could represent an important target to increase cancer sensibility to chemotherapeutics. This possibility has been indirectly investigated by Zhan and coll. [201] in NSCLC cell lines knockdown for KEAP1, the Nrf2 inhibitor. In these cells, the moderate increase of Nrf2 level was associated with the increase of PPARγ and sensibility to cytotoxic effect of arsenic trioxide (As(2)O(3)), etoposide, and doxorubicin.

Recently, another beneficial effect of PPARγ/Nrf2 activation in cancer cells has been reported in a rat model of paclitaxel-induced neuropathic pain Rosiglitazone has been shown to revert the downregulation of PPARγ occurring in the spinal cord of rats showing paclitaxel-induced neuropathic pain, and reduce the pain, probably through the increased expression of Nrf2/HO-1 [103].

## 4. Conclusions

Starting from their classification as members of Nuclear Receptor superfamily, PPARs have been the subject of increasing scientific interest and studies leading to the characterisation of the roles played by the three isoforms in modulating different cell metabolisms and functions in normal cells. Based on the multiplicity of their physiological activities, PPARs have been demonstrated to be able to influence the different phases of carcinogenic process and some cancer cell characteristics, including growth, death, invasivity and resistance to anticancer therapy.

The fact that PPAR ligands include a wide variety of natural substances, as dietary fatty acids and natural antioxidants, makes PPAR activation an attractive and more save approach to selectively target cancer cells.

## Figures and Tables

**Table 1:** Tissue distribution and functions of peroxisome proliferator-activated receptors (PPARs) in lipid and carbohydrate metabolisms.

PPARα Refs. [10,11,12]	liver	↑ Oxidation of fatty acids, ketogenesis, lipid homeostasis
	heart	↑ Oxidation of fatty acids ↓ Glucose uptake
	kidney	↑ Oxidation of fatty acids
	brown adipose tissue	↑ Oxidation of fatty acids
PPARβ/δ Refs. [11,12]	ubiquitous	↑ Oxidation of fatty acids + branched-chain amino acid
PPARγ Refs. [11,13]	white adipose tissue brown adipose tissue	↑ Adipogenesis, glucose homeostasis
	immune cells	↑ Antigen uptake and activation, lipid metabolism, insulin sensitization
	skeletal muscle	↑ Insulin sensitization

**Table 3:** Main antioxidant genes under transcriptional control of PPARs.

Gene	Function	Species	References
Catalase	Decomposition of H_2_O_2_	Human, mouse, rat	[105,106,107]
Heme Oxygenase-1	Induction of DNA binding activities of transcription factors involved in response to oxidative stress.	Mouse	[108,109]
Glutathione Peroxidase 3	Reduction of hydrogen peroxide and related lipid hydroperoxides.	Human	[110]
Superoxide Dismutase	Conversion of two superoxide anions to oxygen and hydrogen peroxide	Mouse	[111]
Thioredoxin	Redox active protein containing two cysteine residues	Human	[112]
CD36	Scavenger receptor for oxidized lipids	Human	[113]
Uncoupling Protein	Modulation of free radical production in mitochondria	Rat	[114,115,116]

**Table 4:** PPAR expression in some cancers in comparison with corresponding normal cells/tissues.

PPAR Isoform	Cancer Type	Change in the Expression	References
γ	Colorectal Carcinoma (human specimens)	Decrease	[157,158]
	Gastric Carcinoma (human specimens)	Decrease	[159]
	Nonmedullary Thyroid Carcinoma (human specimens)	Decrease	[160]
	Cervical Carcinoma (human specimens)	Decrease	[161]
	Esophageal Cancer +(human specimens)	Decrease	[162]
	Prostate Carcinoma (human specimens)	Increase	[163]
	Colorectal Carcinoma (human specimens)	Decrease	[164]
α	Colorectal Carcinoma (CaCo-2 cells)	Increase	[165]
	Ampullary Cancer (human specimens)	Increase	[166]
	Lung Cancer(mouse model)	Decrease	[167]
β/δ	Prostate Carcinoma (human specimens; DU145, PC3, LNCAP, VCAP, C4-2, 22RV1 cell lines)	Decrease	[168]
	Melanoma (human specimens; A375 cell line)	Increase	[169]
	NSCLC (human specimens; H358, H441, H23, A549 cell lines)	Increase	[170]
	Thyroid Cancer (human specimens)	Increase	[171]
	Ovarian Cancer (mouse model)	Increase	[172]
	Colorectal Carcinoma (human specimens)	Increase	[164]
